# Characterization of Nanoemulsions Stabilized with Different Emulsifiers and Their Encapsulation Efficiency for Oregano Essential Oil: Tween 80, Soybean Protein Isolate, Tea Saponin, and Soy Lecithin

**DOI:** 10.3390/foods12173183

**Published:** 2023-08-24

**Authors:** Siqi Zhao, Ziyi Wang, Xuefei Wang, Baohua Kong, Qian Liu, Xiufang Xia, Haotian Liu

**Affiliations:** College of Food Science, Northeast Agricultural University, Harbin 150030, China; zhaosiqi0121@163.com (S.Z.); wangzy711@126.com (Z.W.); neauwangxuefei@163.com (X.W.); kongbh63@hotmail.com (B.K.); liuqian@neau.edu.cn (Q.L.); xiaxiufang@neau.edu.cn (X.X.)

**Keywords:** nanoemulsion, natural emulsifier, stability, oregano essential oil, encapsulation efficiency

## Abstract

The use of the appropriate emulsifier is essential for forming a stable nanoemulsion delivery system that can maintain the sustained release of its contents. Health concerns have prompted the search for natural biopolymers to replace traditional synthetic substances as emulsifiers. In this study, an oregano essential oil (OEO) nanoemulsion-embedding system was created using soybean protein isolate (SPI), tea saponin (TS), and soy lecithin (SL) as natural emulsifiers and then compared to a system created using a synthetic emulsifier (Tween 80). The results showed that 4% Tween 80, 1% SPI, 2% TS, and 4% SL were the optimal conditions. Subsequently, the influence of emulsifier type on nanoemulsion stability was evaluated. The results revealed that among all the nanoemulsions, the TS nanoemulsion exhibited excellent centrifugal stability, storage stability, and oxidative stability and maintained high stability and encapsulation efficiency, even under relatively extreme environmental conditions. The good stability of the TS nanoemulsion may be due to the strong electrostatic repulsion generated by TS molecules, which contain hydroxyl groups, sapogenins, and saccharides in their structures. Overall, the natural emulsifiers used in our study can form homogeneous nanoemulsions, but their effectiveness and stability differ considerably.

## 1. Introduction

Natural preservatives are now more widely used in the food industry because of rising consumer health demands. In this regard, essential oils and their extracts, which are a type of plant-derived natural preservative, are antimicrobial, antioxidant, and nontoxic [1]; thus, they are considered alternatives to synthetic preservatives, such as nitrates, sorbates, and sulfites. Oregano essential oil (OEO) has been considered as GRAS (generally recognized as safe) according to the FDA (Food and Drug Administration), which is derived from *Origanum vulgare* L., an endemic shrub of the *Lamiaceae* family. Carvacrol, p-cymene, and c-terpinene, as main ingredients, give OEO relatively strong antimicrobial properties [2,3]. Dávila-Rodríguez et al. [4] reported that OEO exhibited the most effective antibacterial ability compared with cinnamon essential oil and rosemary essential oil. Similarly, Liu et al. [5] concluded that oregano essential oil could inhibit biofilm formation at a lower concentration than clove essential oil. In addition, numerous other studies have confirmed the excellent antibacterial property of OEO [6,7]. On the other hand, OEO has certain advantages in terms of cost among various essential oils [8]. However, OEO possesses limited applications due to several factors, including high volatility, low water solubility, thermal instability, strong aromatic smell, and sensitivity to environmental stress. Moreover, such unstable agents are susceptible to protein, fat, and other components in food products, resulting in a reduction in their antibacterial activities [9].

To mitigate these limitations, encapsulation techniques have been extensively employed to enhance the benefits of hydrophobic essential oils. Oil-in-water (O/W) nanoemulsions serve as nanoscale encapsulation systems, consisting of two phases that are mutually immiscible, with an average particle size ranging from 20 to 500 nm [10]. By facilitating the interaction between essential oils and microorganisms, augmenting the solubility and absorption of essential oils, and enhancing their release properties, the utilization of nanoemulsions can effectively overcome the drawbacks associated with essential oils [11]. Nevertheless, nanoemulsions are thermodynamically unstable and typically exhibit the phenomena of creaming, flocculation, aggregation, and Ostwald ripening over time [12]. To combat these undesirable phenomena, emulsifiers are constantly required to maintain the stability and uniformity of the system [13]. Currently, most nanoemulsions in practical production are stabilized with synthetic emulsifiers, such as polyoxyethylene sorbitan esters (the Tween family). Tween 80 (T80), the most commonly used synthetic emulsifier, has a hydrophilic–lipophilic balance of 15.0, which allows it to spread relatively easily at the oil–water interface and demonstrate good emulsification effects [14]. However, synthetic emulsifiers may pose a health risk; therefore, researchers are increasingly focusing on natural emulsifiers that are both safe and harmless [15].

Generally, natural emulsifiers include proteins (soybean protein isolate (SPI), whey protein isolate, zein protein, etc.), phospholipids (soy lecithin (SL), etc.), and saponins (tea saponin (TS), quillaja saponin, etc.), all of which have been shown to have emulsifying abilities [16,17,18]. There may be different mechanisms for forming nanoemulsions with different emulsifier types. The appropriate emulsifier should be selected according to specific conditions. Therefore, it is necessary to compare the characterization and stability of nanoemulsions prepared with different emulsifiers under similar conditions. In this study, each natural emulsifier (SPI, TS, or SL) was used to prepare nanoemulsions and was compared to the synthetic emulsifier (T80).

SPI is a rigid globular protein that consists of glycinin and β-conglycinin and is one of the most widely studied protein emulsifiers with high emulsifying activity and surface properties. SPI can be quickly adsorbed onto oil droplet surfaces, reducing interfacial tension and forming a protective film around the droplets to prevent aggregation. Xu et al. [19] confirmed that stable nanoemulsions with droplet sizes below 200 nm could be efficiently stabilized by SPI, 7S, and 11S proteins. TS is a pentacyclic triterpenoid extracted from the *Camellia oleifera* seed meal of camellia plants, and it has been proven to be safe, environmentally friendly, and easily decomposed by microorganisms [20]. Additionally, TS is a type of agricultural by-product that has a more sustainable source than other natural emulsifiers. According to previous reports, the surface activity of TS is attributed to hydrophilic glycosyl and hydrophobic aglycons in its structure. Deng et al. [17] successfully encapsulated silymarin with TS, and the mechanism of TS as an emulsifier may be due to the hydroxyl groups, sapogenins, and saccharides in its structure. SL is a natural mixed emulsifier derived from the cell membranes of soybeans, and it comprises phosphatidylcholine, phosphatidylethanolamine, phosphatidylserine, phosphatidylinositol, phosphatidylglycerol, and other phospholipid derivatives. SL can be used as a healthcare product because it can regulate blood lipids, delay aging, etc. [21]. More importantly, SL contains hydrophobic alkyl side chains and hydrophilic groups, which endow it with amphiphilicity and reduce the interfacial tension between phases [22]. In recent years, SL has frequently been used as a natural emulsifier to stabilize nanoemulsions, which exhibited good emulsifying properties, according to Nash et al. [18], Mehmood et al. [23], and Sandoval et al. [24].

Based on the abovementioned implementability, we prepared nanoemulsions with these four emulsifiers (three natural emulsifiers and one synthetic emulsifier) to investigate the influence of emulsifier type and concentration on the characteristics of nanoemulsions. Subsequently, specific concentrations of each emulsifier were screened to further evaluate the effects of emulsifier types on the stability of nanoemulsions. Moreover, OEO was encapsulated by the nanoemulsion carriers, and the influence of environmental conditions (pH, ionic strength, and heat) on OEO nanoemulsions was evaluated to further verify whether the nanoemulsions encapsulated with OEO could still maintain a stable state during actual processing. This study aims to compare the effects of different emulsifiers on the formation and stability of nanoemulsions in order to provide a theoretical basis for broadening the application of natural emulsifiers. This study also aims to provide new ideas and solutions for the rational construction of nanoemulsion delivery systems.

## 2. Materials and Methods

### 2.1. Materials

T80 was purchased from the Solabio Corporation (Beijing, China). SPI, TS, SL, medium chain triglyceride (MCT) oil, and OEO were obtained from Yuan Ye Biological Technology Co., Ltd. (Shanghai, China). All other chemical reagents used in this study were of analytical grade.

### 2.2. Nanoemulsion Preparation

O/W nanoemulsions were prepared by homogenizing a 5% (*v*/*v*) oil phase (MCT) with a 95% (*v*/*v*) aqueous phase according to Zou et al. [25], with a slight modification. The aqueous phases required for the formation of nanoemulsions were prepared by dispersing various concentrations (0.5–8% *w*/*v*) of each emulsifier (T80, SPI, TS, and SL) in deionized water. Thereafter, the mixture was stirred continuously at room temperature (~25 °C) for 2 h and then stored at 4 °C overnight to ensure complete hydration. To form a coarse emulsion, the oil and aqueous phases were blended in a high-speed homogenizer (IKA, Staufen, Germany) for 2 min at a speed of 14,000 rpm. Subsequently, the coarse emulsions were passed through a high-pressure microfluidizer (AFM-3, ATS Engineering Limited, Cambridge, Ontario, Canada) at 50 MPa for two cycles to obtain fine nanoemulsions.

### 2.3. Measurement of the Droplet Size, Polydispersity Index (PDI), and Zeta Potential

Based on the methodology of Lotfy et al. [26], the droplet size, PDI, and zeta potential of the nanoemulsions were measured using a dynamic light scattering instrument (ZetasizerNano-ZS90, Malvern, UK). To avoid multiple scattering effects, each nanoemulsion sample was diluted 100-fold with deionized water. The refractive indices of the oil and water were set at 1.47 and 1.33, respectively. Each measurement was conducted in triplicate at 25 ± 2 °C.

### 2.4. Super-Resolution Micromorphology

Microstructural analysis was conducted using a Deltavision OMX SR super-resolution microscope (GE Co., Boston, Massachusetts, USA) to elucidate the particle size and distribution of the nanoemulsions. Based on the work of Liu et al. [27], 1 mL of the nanoemulsions was mixed with 20 μL of Nile red (0.1%, *w*/*v*) and then left in the dark for 30 min after vortex mixing. The stained nanoemulsion samples (5 μL) were deposited on a concave slide with a coverslip. The samples were stored at 4 °C for 12 h before observation.

### 2.5. Rheological Properties

The apparent viscosity of the nanoemulsions was determined at 25 °C using a HAAKE MARS60 modular rotary rheometer (Thermo Fisher Scientific, Shanghai, China) according to the method described by Wang et al. [28]. First, 3 mL of fresh nanoemulsions was loaded on the steel parallel plate of the rheometer (geometry diameter: 40 mm; gap size: 1.0 mm). Thereafter, the flow curves were obtained at a shear rate range of 0.1–100 s^−1^.

### 2.6. Nanoemulsion Stability

#### 2.6.1. Centrifugal Stability

The centrifugal stability of the nanoemulsions was determined by measuring the particle size and zeta potential before and after centrifugation (4500× *g*, 15 min) and then measuring the centrifugal stability constant (Ke).

To obtain the Ke values, the absorption values of the 100-fold-diluted nanoemulsion were evaluated at 500 nm using a spectrometer according to the method reported by Qi et al. [29], with minor modifications. These absorption values were denoted as A1. Subsequently, each nanoemulsion sample was centrifuged at 4500× *g* for 15 min and then collected to determine their absorbance at 500 nm, which was recorded as A2. Finally, Ke was calculated using the following equation:

Ke (%) = (A1 **−** A2)/A1 × 100 (1)

A low Ke value indicates that the nanoemulsion is stable.

#### 2.6.2. Storage Stability

The nanoemulsions prepared with different emulsifiers were stored at 4 °C and 25 °C for 15 days. Changes in the particle size and zeta potential were assessed every three days for 15 days (on days 0, 3, 6, 9, 12, and 15).

#### 2.6.3. Oxidative Stability

To evaluate their oxidative stability, the nanoemulsions were placed in centrifuge tubes and kept in a constant-temperature incubator at 50 °C for 7 days to accelerate oxidation. The concentrations of primary oxidation products (hydroperoxides) and secondary oxidation products (thiobarbituric acid-reactive substances (TBARS)) in the nanoemulsions were determined using the methodology of Li et al. [30].

To measure the hydroperoxides, 1 mL of oxidated nanoemulsions was mixed with 3 mL of isooctane/2-propanol (3:1, *v*/*v*), and the mixture was vortexed for 1 min. Subsequently, the mixture was centrifuged at 2000 *g* for 5 min to collect the organic phase. The supernatant (200 μL) was mixed with 2.8 mL of methanol/1-butanol (2:1, *v*/*v*), 50 μL of NH_4_SCN (3.94 M), and 50 μL of 0.132 M BaCl_2_/0.144 M FeSO_4_ (1:1, *v*/*v*). After 20 min of reaction, the absorbance of each mixture was determined at 510 nm using a spectrophotometer with methanol/1-butanol as a blank. The hydroperoxide concentration was obtained using a standard curve prepared with cumene hydroperoxide.

To measure TBARS, 1 mL of the nanoemulsions was mixed with 2 mL of a TBA solution and vortexed for 1 min. Thereafter, the mixtures were placed in a boiling water bath for 15 min, cooled to room temperature, and then mixed with 3 mL of chloroform under stirring. Subsequently, the mixtures were centrifuged at 3000× *g* for 15 min, and then the absorbance of the supernatant was determined at 532 nm using a spectrophotometer.

### 2.7. OEO-Nanoemulsion Preparation

OEO-nanoemulsions were prepared by homogenizing a 5% (*v*/*v*) oil phase with a 95% (*v*/*v*) aqueous phase [25,31]. The aqueous phase was prepared using the same method described in Section 2.2, but there were some differences in the composition of the oil phase. Briefly, MCT and OEO were mixed in a ratio of 1:1 (*v*/*v*) and stirred overnight to form the oil phase. Thereafter, the oil and aqueous phases were mixed in a high-speed homogenizer for 2 min at a speed of 14,000 rpm to produce coarse emulsions. Finally, the coarse emulsions were homogenized using a high-pressure microfluidizer at 50 MPa for two cycles. The details of the composition of the nanoemulsion samples are exhibited in Table 1.

Emulsifier concentration refers to the mass concentration in the aqueous phase. The concentration of aqueous phase, MCT-oil, and OEO refer to the volume concentration in nanoemulsions.

### 2.8. Encapsulation Efficiency

The encapsulation efficiency was assessed using the approach of Davila et al. [13] with a slight modification. To determine the encapsulation efficiency, ethanol, n-hexane, and the OEO-nanoemulsions were mixed in a ratio of 2:3:1 (*v*/*v*) with gentle hand-shaking. Subsequently, the absorbance of free OEO dissolved in the supernatant was measured at 255 nm using a UV–Vis spectrophotometer. Finally, the free OEO concentration was calculated using a standard curve constructed with a series of OEO solutions. The encapsulation efficiency was then obtained using the following equation:

EE (%) = (Total OEO amount (g) − Free OEO amount(g))/Total OEO amount (g) × 100(2)

### 2.9. Environmental Stability of the OEO Nanoemulsion

#### 2.9.1. pH Stability

To evaluate the effect of pH on the OEO-nanoemulsions stabilized with different emulsifiers, 0.1 M HCl and 0.1 M NaOH were used to adjust the pH of the samples to values ranging between 5 and 9. Thereafter, changes in the OEO-nanoemulsions were examined by measuring their particle size, zeta potential, encapsulation efficiency, and appearance. The fresh nanoemulsions after adjusting were stored at 4 °C for 12 h before analysis. Each measurement was performed at 25 ± 2 °C.

#### 2.9.2. Ionic Strength Stability

The ionic strength of the OEO-nanoemulsions was adjusted to 100–500 mM by adding different quantities of NaCl powder. Thereafter, the adjusted nanoemulsions were stored at 4 °C for 12 h prior to particle size, zeta potential, encapsulation efficiency, and appearance measurements. The test was conducted at 25 ± 2 °C.

#### 2.9.3. Thermal Stability

To assess their thermal stability, the OEO-nanoemulsions were placed in water baths at temperatures of 60–90 °C for 30 min before being cooled to room temperature. Finally, their particle size, zeta potential, encapsulation efficiency, and appearance were measured.

### 2.10. Statistical Analyses

Three batches of nanoemulsions were prepared, and all measurements were conducted in triplicate. The values were reported as mean ± standard deviation (SD) and were analyzed using the general linear model procedure from the Statistix 8.1 software package (Analytical Software, St. Paul, MN, USA). Analysis of variance (ANOVA) was used with Tukey’s multiple comparison tests to determine the significance among the samples (*p* < 0.05).

## 3. Results and Discussion

### 3.1. Droplet Size and PDI

Droplet size, which can also affect physicochemical stability, is the most basic characteristic for describing nanoemulsions [32]. As shown in Table 2, the nanoemulsion droplet size decreased rapidly as the T80, TS, and SL emulsifier concentrations increased from 0.5% to 4%, and then it decreased relatively slowly as the emulsifier concentrations increased from 4% to 8%. This phenomenon may be attributed to two stages of nanoemulsion formation. Initially, low emulsifier concentrations (0.5–4%) made more emulsifier molecules available to cover the surfaces of newly created oil droplets, resulting in smaller droplets. However, once the emulsifier concentrations exceeded 4%, there were sufficient emulsifier molecules around the droplet surfaces. Thus, the droplet size maintained an almost constant value even though the emulsifier concentration increased at this stage [33]. Similar findings were reported by Arancibia et al. [34] and Zhu et al. [35], who concluded that the dependence of nanoemulsion droplet size on emulsifier (T80, TS, and SL) levels can be divided into two regimes: a “surfactant-limited” regime and a “surfactant-rich” regime.

The droplet size of the SPI nanoemulsion exhibited a different trend from those of the above three nanoemulsions. Overall, it first decreased and then rapidly increased as the SPI concentration increased, as shown in Table 2. The droplet size of the SPI nanoemulsion decreased significantly (*p* < 0.05) as the SPI concentration increased from 0.5% to 1%. This is because a low SPI concentration is insufficient to completely cover the entire oil droplet surfaces. When the SPI concentration was increased, the SPI molecules could be adsorbed at the oil−water interface, thereby decreasing the droplet size. However, the droplet size increased significantly (*p* < 0.05) as the SPI concentration increased from 1% to 8%. This phenomenon may be because the excessive free proteins in the aqueous phase aggregate with each other to form submicelles, which could increase the oil droplet size and compromise the stability of the nanoemulsions [36].

Table 2 shows that there was no significant difference in the PDI (*p* > 0.05) as each emulsifier concentration increased. Additionally, the PDI values of each nanoemulsion ranged from 0.16 to 0.21. Generally, it is considered that nanoemulsions with PDI values below 0.25 can exhibit a uniform droplet-size distribution and thus good physical stability [37]. Consequently, it could be concluded that a relatively fine nanoemulsion would be formed with the entire concentration range (0.5–8%) of T80, SPI, TS, and SL. However, there are certain differences in the particle size of emulsions stabilized with different emulsifiers. These differences may be due to several factors, including how quickly the emulsifiers attach to the oil droplet surfaces, their ability to reduce interfacial tension, and their effects on interfacial rheology [35].

### 3.2. Zeta Potential

The zeta potential is a measure of the surface charge density of nanoemulsion droplets [38], and it was evaluated in this study to further explore the properties of the interfaces formed by the four emulsifiers. Table 2 presents the zeta potential values. All the nanoemulsions exhibited a negative electrical charge, which may be due to the electrical charge of the emulsifier molecules. Although T80 is a nonionic surfactant, the zeta potential of the T80 nanoemulsion was a negative charge. This may be attributed to the presence of free fatty acid impurities in the surfactant or the ability of hydroxyl ions to be adsorbed from water to the oil droplet surfaces [39]. SPI nanoemulsions have a negative electrical charge because of the negatively charged amino acid residues on the protein surfaces [40]. Similarly, TS nanoemulsion droplets have a negative surface potential because of the presence of carboxylic acid groups on the absorbed saponin molecules [35]. Phosphatidylcholine, the main component of SL, would impart a negative charge to nanoemulsion droplets because it is a zwitterionic lipid [41].

Furthermore, as depicted in Table 2, the change in the absolute zeta-potential value corresponds to the abovementioned trend in droplet size in that the nanoemulsions with relatively small droplet sizes had relatively high net charges. Similar findings were reported by Hu et al. [36]. However, this correspondence was not noticeable in the T80 nanoemulsions. This could be because T80 nanoemulsions are mainly stabilized by steric hindrance [39], while SPI, TS, and SL nanoemulsions prevent aggregation through electrostatic repulsion [42]. Generally, it is considered that absolute zeta potential values of >30 mV could ensure nanoemulsion stability against aggregation and coalescence [43]. In this case, nanoemulsions stabilized with SPI and TS would therefore be highly stable because of the strong electrostatic repulsion between the droplets.

### 3.3. Super-Resolution Microscopy

**Figure 1:**
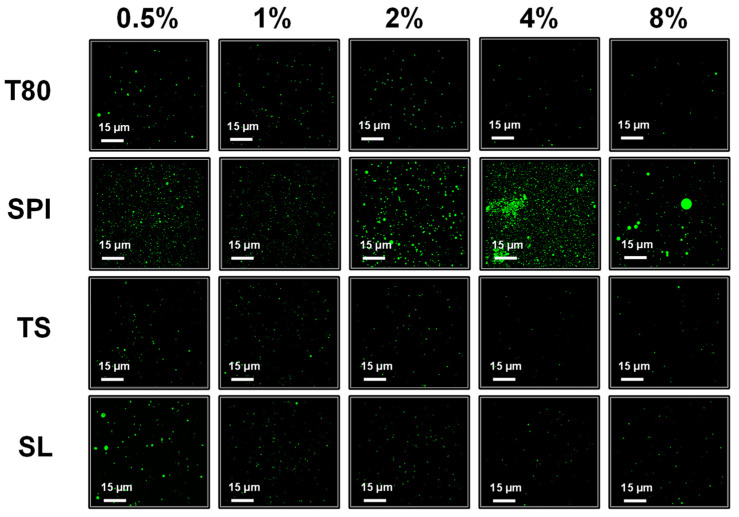
Super-resolution microscopic images of the nanoemulsions prepared with different emulsifier types and concentrations (0.5–8%, *w*/*v*). The green dots represent oil droplets of nanoemulsions. T80: Tween 80; SPI: soybean protein isolate; TS: tea saponin; SL: soy lecithin.

Super-resolution microscope imaging was conducted to observe the influence of emulsifier type and concentration on the droplet distribution and morphology of the nanoemulsions. Figure 1 shows that the green oil droplets have spherical shapes, implying that the oil droplets were completely encapsulated inside the nanoemulsions [30].

When the emulsifier concentration was 0.5%, the T80, TS, and SL nanoemulsions contained numerous large particles and had a relatively nonuniform droplet distributions. When the emulsifier concentration exceeded 0.5%, the droplet size reduced significantly, and the distribution became more uniform. When the emulsifier concentration exceeded 4%, the oil droplets were almost invisible through the super-resolution microscope. Contrarily, the smallest oil droplets and the most uniform droplet distribution were observed in the sample prepared with 1% SPI. However, as the SPI concentration further increased from 1% to 8%, the droplet size gradually increased, and at high SPI concentrations (4%–8%), the droplets exhibited merging behavior. These results are consistent with the change in droplet size, further illustrating that different emulsifiers have different effects on the formation of nanoemulsions.

### 3.4. Rheological Properties

**Figure 2:**
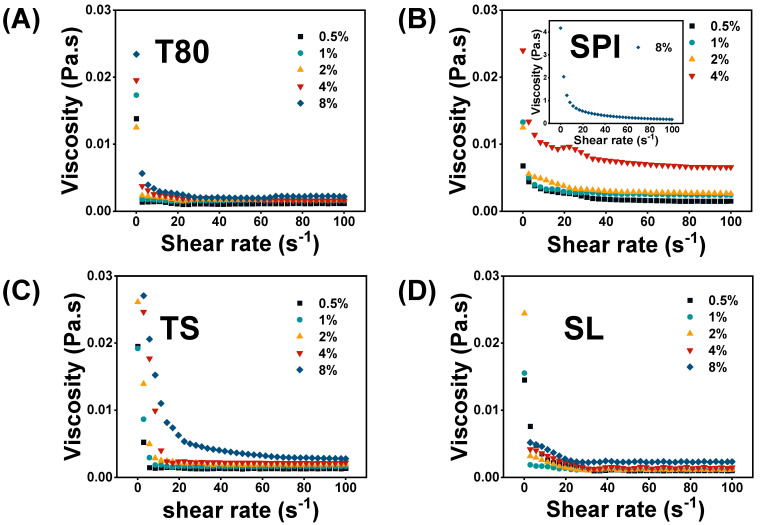
Apparent viscosities of the nanoemulsions stabilized with (**A**) T80, (**B**) SPI, (**C**) TS, and (**D**) SL.

Rheology plays an important role in the characterization of interfacial films, which are related to the stability of nanoemulsions [44]. As displayed in Figure 2, all the investigated nanoemulsions exhibited a gradual decrease in viscosity within the shear rate range of 0.1–100 s^−1^, demonstrating shear-thinning behavior, a common feature of conventional emulsions. At low shear rates (0.1–20 s^−1^), the viscosity of all the samples decreased sharply, which may be due to the deflocculation of the oil droplets and the deformation of the flocs under the shear field. Conversely, at high shear rates (20–100 s^−1^), the viscosity did not change significantly, demonstrating the properties of pseudoplastic fluids. This can be attributed to the flocs splitting into single droplets and then maintaining a relatively stable state [45].

Furthermore, Figure 2 clearly shows that the viscosity of the nanoemulsions increased as the emulsifier concentration increased, particularly for the SPI-stabilized emulsions. This dose-dependent relationship may be due to two factors: (1) the thickening effects of emulsifiers cause excessive emulsifier molecules to migrate into the aqueous phase, thereby increasing the viscosity of the nanoemulsions, and (2) apparent viscosity is related to the droplet size of nanoemulsions (that is, the smaller the droplet size, the larger the specific surface area of the nanoemulsions, and the easier the interaction between oil droplets, which ultimately increases the apparent viscosity) [46]. Interestingly, the viscosity of the SPI nanoemulsion increased by nearly two orders of magnitude when the SPI concentration increased to 8% (Figure 2B). Such a large increase in viscosity may be attributed to the gel network formed by unabsorbed proteins in the continuous phase, which decreased the fluidity of the nanoemulsions. Similar findings were reported by Kadiya et al. [47], who revealed that the significant increase in the apparent viscosity of nanoemulsions stabilized with whey protein isolate and pectin was mainly due to the excessive emulsifiers in the aqueous phase.

The T80-stabilized nanoemulsions maintained a low apparent viscosity throughout the concentration range, unlike the other nanoemulsions (Figure 2A). This may be attributed to the thin interfacial film produced by T80. As the shear rate increased, the internal structure of the nanoemulsion system was easily destroyed, resulting in a decrease in viscosity. According to Stoke’s law, a high viscosity could generally retard the migration rate of oil droplets, thereby improving the stability of nanoemulsions [48]. Therefore, it could be inferred that the SPI, TS, and SL nanoemulsions were more stable than the T80 nanoemulsions. Other factors, however, such as droplet size and certain environmental conditions, can also influence the stability of nanoemulsions.

### 3.5. Nanoemulsion Stability

Based on the aforementioned results, 4% T80, 1% SPI, 2% TS, and 4% SL were chosen to form nanoemulsions in order to analyze the stability of the nanoemulsions under different conditions.

#### 3.5.1. Centrifugal Stability

**Figure 3:**
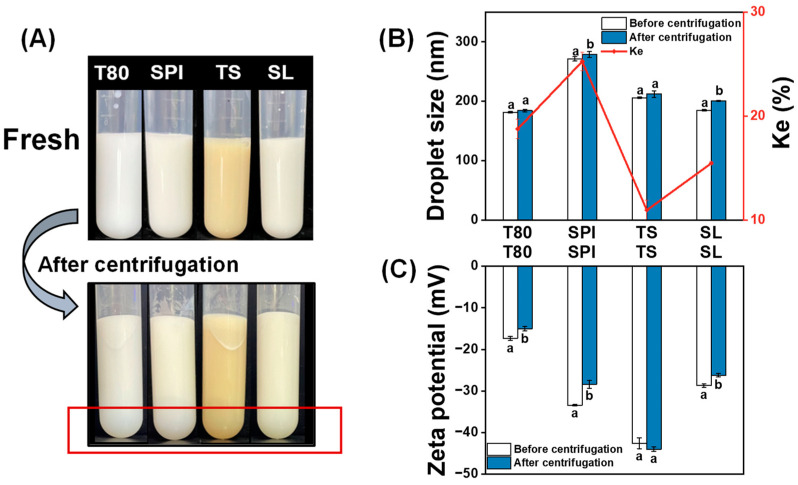
(**A**) Appearance, (**B**) droplet size, centrifugal stability constant (Ke), and (**C**) zeta potential of the nanoemulsions prepared with different emulsifiers (T80, SPI, TS, and SL) before and after centrifugation. The letters a–b indicate significant differences between the same nanoemulsion before and after centrifugation (*p* < 0.05).

As shown in Figure 3A, the bottom of each centrifuge tube became clearer after centrifugation because the oil droplets floated during the high-speed centrifugation. To obtain a more specific description of how the nanoemulsions changed under this extreme condition, the Ke value, which could quantify the intuitive appearance, was used to determine the turbidity of the nanoemulsions before and after centrifugation. Generally, when the Ke value is low, the nanoemulsion is considered to be relatively stable [29]. Figure 3B shows that the TS nanoemulsion exhibited the lowest Ke value among the tested samples because of the strong electrostatic repulsion between its oil droplets (Figure 3C), which made it able to resist the centrifugal force. As shown in Figs. 3B and 3C, the droplet size and zeta potential of the TS nanoemulsion did not exhibit significant changes before and after centrifugation (*p* > 0.05). Conversely, the SPI nanoemulsion had the highest Ke value and the most noticeable changes in droplet size and zeta potential. This may be attributed to the high gravitational migration ability of large droplets [49].

Generally, the application of centrifugal force to nanoemulsions results in instability phenomena, such as precipitation, phase separation, and creaming, as well as a sharp increase in the droplet size of the nanoemulsion to the micron level [50]. In this study, the nanoemulsions prepared with different emulsifiers did not exhibit these phenomena after the application of a strong centrifugal force, proving that the investigated nanoemulsions, particularly the TS nanoemulsion, had good physical stability.

#### 3.5.2. Storage Stability

**Figure 4:**
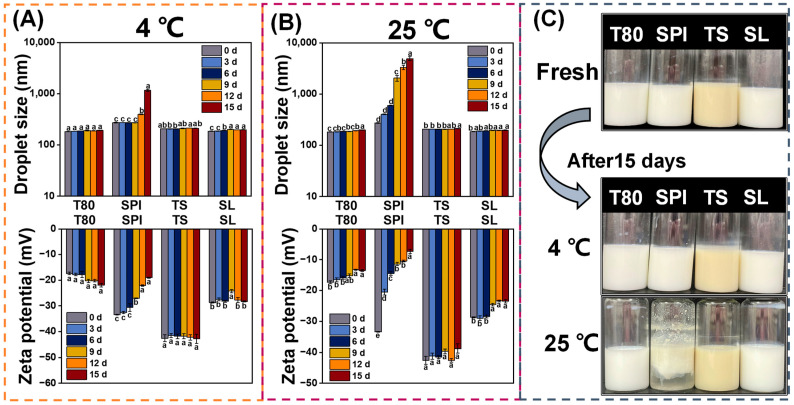
Effects of storage time (0–15 days) on the (**A**,**B**) droplet size, zeta potential, and (**C**) appearance of the nanoemulsions prepared with different emulsifiers (T80, SPI, TS, and SL) at 4 °C and 25 °C. The letters a–d indicate the significant differences for the same nanoemulsion at different storage times (*p* < 0.05).

The storage stability of nanoemulsions is an essential indicator of the shelf life of products in practical applications [51]. Figure 4 shows the droplet size, zeta potential, and appearance of the different stabilized nanoemulsions stored at 4 °C and 25 °C for 15 days.

The T80, TS, and SL nanoemulsions were discovered to exhibit no significant difference in zeta potential and only a slight increase in droplet size during the 15-day storage at 4 °C (Figure 4A). Moreover, there was no noticeable change in appearance, as shown in Figure 4C. However, the SPI nanoemulsion eventually exhibited a different phenomenon, with a sharp increase in droplet size and zeta potential from the ninth day at 4 °C. Specifically, after 15 days, the droplet size of the SPI nanoemulsion was threefold larger than that of the fresh nanoemulsion, and its zeta potential decreased nearly twofold. This instability may be ascribed to the aging of austenite and the variations in protein conformation, which cause the formation of intermolecular hydrogen and hydrophobic bonds, inducing oil droplet aggregation [30,52]. Compared to the SPI molecules, the T80, TS, and SL molecules were more fairly efficient at preventing droplet flocculation or aggregation. This is because T80, TS, and SL were able to produce smaller oil droplets (Figure 1), which could help to decrease the rate of gravitational separation [33].

As shown in Figure 4B, when the nanoemulsions were stored at 25 °C, there were noticeable variations in their droplet sizes and zeta potentials. Additionally, when the appearance of the SPI nanoemulsion was observed, some instability phenomena, including flocculation, creaming, and phase separation, were discovered. Accordingly, it was confirmed that the nanoemulsions were more stable at 4 °C than at 25 °C. This may be explained by the fact that the droplet collision rate and frequency were relatively high at 25 °C, which may have caused an increase in droplet size [53]. These findings are similar to those of Tian et al. [54], who concluded that the higher the storage temperature, the longer the storage time, and the larger the particle size.

#### 3.5.3. Oxidative Stability

**Figure 5:**
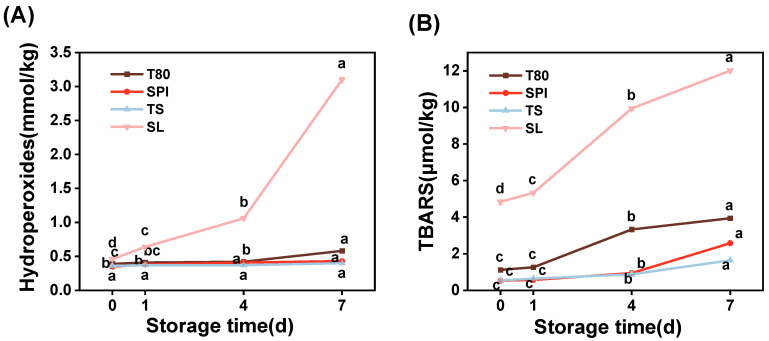
Formation of (**A**) lipid hydroperoxides and (**B**) thiobarbituric acid-reactive substances (TBARS) in the nanoemulsions stabilized with different emulsifiers (T80, SPI, TS, and SL) at 50 °C for 7 days. The letters a–d indicate the significant differences for the same nanoemulsion at various storage times (*p* < 0.05).

Oxidative stability was determined by monitoring the formation of primary (hydroperoxides) and secondary (TBARS) reaction products throughout the storage process [55]. The primary and secondary products in all the nanoemulsion samples gradually increased with prolonged storage time, indicating that lipid oxidation occurred (Figure 5). Figure 5A the SL nanoemulsion had a significantly higher increase in hydroperoxide content than the other nanoemulsions. A significant change in hydroperoxide content appeared in the T80 and SPI nanoemulsions from the fourth day of storage. However, there was no significant change in the hydroperoxide content in the TS nanoemulsion (*p* > 0.05). Moreover, the trends of the TBARS content in the nanoemulsions were remarkably similar to those of the hydroperoxide content. The content and formation rate of TBARS in the different samples decreased in the following manner: SL nanoemulsion > T80 nanoemulsion > SPI nanoemulsion > TS nanoemulsion.

This difference is related to the nature of the emulsifiers. The SL nanoemulsion depicted the worst oxidative stability because SL is susceptible to lipid oxidation, autoxidation, and photosensitized lipid oxidation in nanoemulsions, according to Arancibia et al. [35]. Furthermore, the SL amount was relatively high, which increased the oxidation level. For the T80 nanoemulsion, the high content of oxidation products may be due to the polyether-based hydrophilic head group, which could be easily oxidized. Additionally, as the main prooxidants, the transition metals present in the aqueous phase could initially promote the decomposition of lipid hydroperoxides on the oil droplet surfaces into free radicals and then promote the lipid oxidation process [54]. In contrast, proteins and saponins have been confirmed to effectively inhibit lipid oxidation in nanoemulsions. Zhang et al. [56] reported that unabsorbed proteins can scavenge free radicals and chelate metal ions. Thus, these prooxidants can be prevented from being absorbed onto the oil droplets’ surfaces. TS protects against oxidation mainly by scavenging free radicals, which may be because of the hydroxyl group in its molecular structure [17]. These results demonstrate that TS is the most effective emulsifier for enhancing the oxidative stability of nanoemulsions.

### 3.6. Environmental Stability of OEO Nanoemulsions

Based on these results, we attempted to evaluate the stability of the nanoemulsions under different environmental conditions after encapsulating OEO in order to further compare the effectiveness of the different emulsifiers. When SL was used as an emulsifier to encapsulate OEO, no uniform nanoemulsion was formed. We speculated that this anomaly was due to the reaction between the SL molecules and OEO, which diminished the emulsification ability of SL. Therefore, T80, SPI, and TS were chosen to prepare OEO-nanoemulsions in the next study.

#### 3.6.1. pH Stability

**Figure 6:**
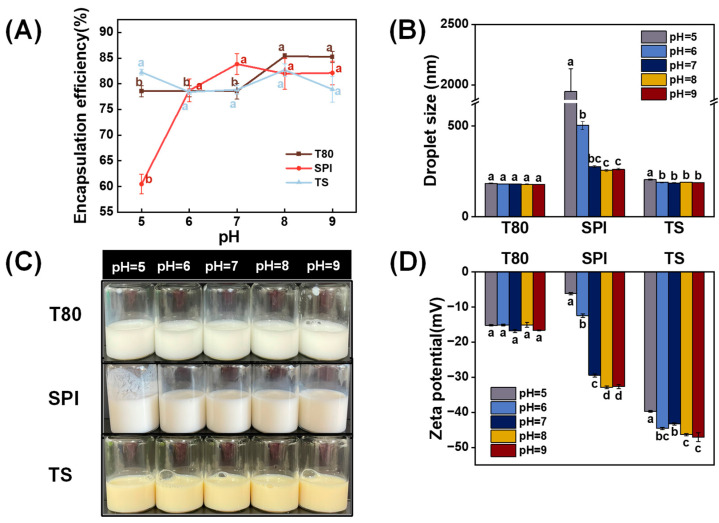
Effects of pH (5–9) on the (**A**) encapsulation efficiency, (**B**) droplet size, (**C**) appearance, and (**D**) zeta potential of the OEO-nanoemulsions stabilized with different emulsifiers (T80, SPI, and TS). The letters a–d indicate the significant differences for the same nanoemulsion at different pH levels (*p* < 0.05).

It is essential to evaluate the effect of pH on nanoemulsions since different pH values have been frequently found in commercial foods in practical applications. The T80 OEO-nanoemulsion exhibited high stability without significant changes in droplet size, zeta potential, and appearance from pH 5 to 9 (Figure 6B–D). This suggests that the T80-coated droplets were primarily stabilized by steric hindrance. Consequently, the influence of pH on the T80 OEO-nanoemulsion was extremely minor. Interestingly, the encapsulation efficiency of the T80 OEO-nanoemulsion slightly improved when the pH value increased to 8 and 9 (Figure 6A). Actually, every droplet in the OEO-nanoemulsion could be considered as a “core-shell” structure. The OEO in nanoemulsion systems was divided into two parts. One part of the OEO was encapsulated in this “core-shell” structure, while the other was free in the aqueous phase. When pH value reached 8–9, hydroxyl ions were adsorbing on the oil–water interface. This may cause an increase in the emulsifying ability of T80, which may allow more OEO to be encapsulated inside the “core-shell” structure, and therefore resulted in efficient OEO encapsulation [57]. A different phenomenon occurred in the SPI and TS nanoemulsions, which were mainly stabilized via electrostatic repulsion. Figure 6 shows that the SPI OEO-nanoemulsion was stable at pH values of 7–9. However, the evidence of instability appeared in the droplet size and appearance of SPI OEO-nanoemulsion at low pH values of 5 and 6. Additionally, the encapsulation efficiency decreased to 60.47% when the pH value was 5 (Figure 6A). The TS OEO-nanoemulsion exhibited greater pH stability than the SPI OEO-nanoemulsion. There was a slight increase in the droplet size of the TS OEO-nanoemulsion at pH 5, but there were no significant variations in the appearance and encapsulation efficiency of the TS OEO-nanoemulsion.

The influence of pH on zeta potential was evaluated to identify the mechanism underlying the instability of the SPI and TS OEO-nanoemulsions in low-pH settings. As displayed in Figure 6D, the absolute zeta potential value of the SPI OEO-nanoemulsion decreased significantly as the pH decreased (*p* < 0.05). This result could be explained by the following reasoning. The larger the absolute zeta potential value, the stronger the mutual repulsion between the nanoemulsion droplets, and the more stable the nanoemulsion. However, when the pH value is close to the isoelectric point of SPI, the electrostatic repulsion is insufficient to overcome attractive forces, and then the droplets tend to aggregate through hydrophobic attraction and van der Waals interaction, resulting in large particles and affecting OEO encapsulation [58]. Similarly, the absolute zeta potential value of the TS OEO-nanoemulsion also decreased significantly as the pH value decreased (*p* < 0.05). This may be due to the protonation of carboxylic acid groups around and below the pK_a_ value of TS [38]. Similar results can be found in a previous study conducted by Yang et al. [59], who evaluated the pH stability of quillaja saponin emulsions. Nevertheless, the lowest absolute zeta potential value of the TS OEO-nanoemulsion was still above 30 mV, implying that TS OEO-nanoemulsions have good pH stability.

#### 3.6.2. Ionic Strength Stability

Investigating the effects of ionic strength on nanoemulsions is also important because commercial foods usually contain different salt levels. Therefore, we added NaCl (100–500 mM) to the nanoemulsions to investigate their stability against ionic strength.

**Figure 7:**
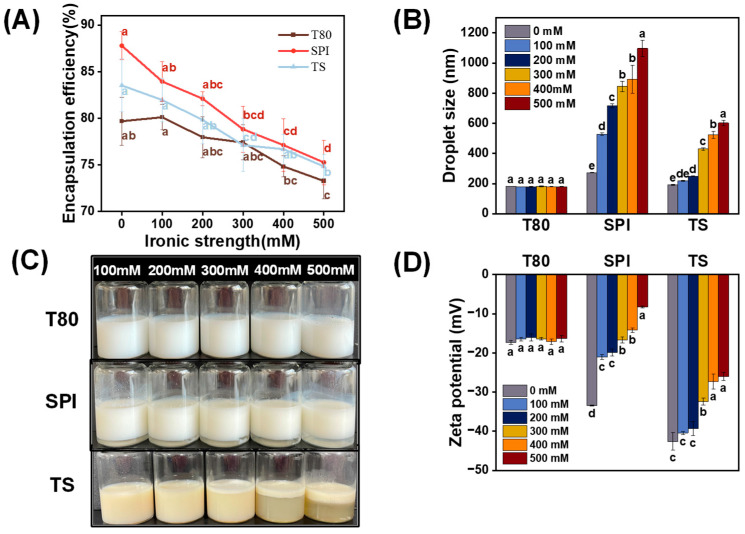
Effects of ionic strength (0–500 mM) on the (**A**) encapsulation efficiency, (**B**) droplet size, (**C**) appearance, and (**D**) zeta potential of the OEO-nanoemulsions prepared with different emulsifiers (T80, SPI, and TS). The letters a–d indicate the significant differences for the same nanoemulsion after different ionic strength treatments (*p* < 0.05).

The droplet size and zeta-potential of the T80 OEO-nanoemulsion did not vary significantly as the ionic strength increased (*p* > 0.05). Additionally, no signs of instability were found through visual observation (Figure 7C). This result may be due to the polyether-based hydrophilic head group of T80, which provided strong steric hindrance. As depicted in Figure 7D, the zeta potential of the SPI OEO-nanoemulsion decreased significantly when more NaCl was added (*p* < 0.05), indicating that ions destroyed the electric double layer of the protein molecules and led to the destruction of the interface layer. This caused the nanoemulsion droplets to aggregate and become unstable, thereby affecting the encapsulation efficiency. Furthermore, an appearance analysis revealed that the SPI nanoemulsion exhibited a stratification phenomenon as the NaCl amount increased (Figure 7C). Similar results were reported by Teo et al. [58], who discovered that whey-protein-isolate-stabilized nanoemulsions became unstable at high salt levels. The variation trend of the TS OEO-nanoemulsion was similar to that of the SPI OEO-nanoemulsion but to a milder degree. Figure 7B–D show that the droplet size, zeta potential, and appearance of the TS nanoemulsion changed slightly at low ionic concentrations (100–200 mM). However, when the salt concentration exceeded 300 mM, the droplet size and zeta potential increased rapidly, and the TS OEO-nanoemulsion formed a white cream layer on top. Additionally, the encapsulation efficiency decreased continuously as the ionic strength increased (*p* < 0.05). This could be attributed to the accumulation of counterions around the charged surface groups, a phenomenon defined as an electrostatic screening effect. At low ionic concentrations, the electrostatic repulsion between the droplets was sufficient to resist aggregation and flocculation. Conversely, at high ionic concentrations, this force could not overcome the van der Waals attraction, resulting in the instability of the TS OEO-nanoemulsion [60].

#### 3.6.3. Thermal Stability

**Figure 8:**
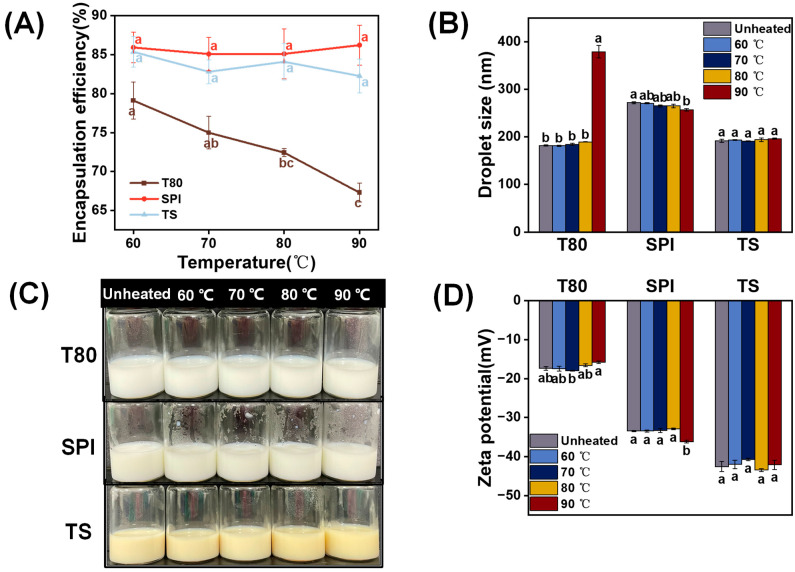
Effects of temperature (60–90 °C) on the (**A**) encapsulation efficiency, (**B**) droplet size, (**C**) appearance, and (**D**) zeta potential of the OEO-nanoemulsions prepared with different emulsifiers (T80, SPI, and TS). The letters a–c indicate the significant differences for the same nanoemulsion after different heat treatments (*p* < 0.05).

Figure 8B,D show that the T80-stabilized OEO-nanoemulsion could remain stable at 60–80 °C. However, when the temperature increased to 90 °C, the droplet size increased steeply, and the absolute zeta potential value slightly decreased. This effect is caused by the temperature being close to the phase inversion temperature of this nonionic surfactant, thereby accelerating the coalescence of the oil droplets [61]. Therefore, owing to the impaired emulsifying ability, the encapsulation efficiency of T80 decreased gradually as the temperature increased. It was discovered that heat treatment did not affect the appearance (Figure 8C) and encapsulation efficiency (Figure 8A) of the SPI OEO-nanoemulsion. Meanwhile, it was observed that as the temperature increased, the droplet size of the SPI OEO-nanoemulsion decreased gradually, and the absolute zeta-potential value increased gradually (Figure 8B,D). As previously reported, SPI can be partially denatured at 90 °C to generate soluble aggregates with a high surface hydrophobicity, which is conducive to its adsorption at the oil–water interface [62]. Similar results were observed in the whey protein isolate nanoemulsions prepared by Teo et al. [58]. The TS OEO-nanoemulsion displayed greater thermal stability than the above two nanoemulsions. Overall, heat treatment had no significant effect on the droplet size, zeta potential, encapsulation efficiency, and appearance of the TS-stabilized OEO-nanoemulsions. This may have been because there was a powerful electrostatic repulsion between the nanoemulsion droplets over the entire temperature range, which is attributed to the carboxylic acid groups in the TS chemical structure. This result agrees with the results reported by Li et al. [63], who confirmed that no signs of destabilization were observed in the quillaja saponin nanoemulsion after temperature treatment.

## 4. Conclusions

In this study, nanoemulsions were prepared with different concentrations of T80, SPI, TS, and SL emulsifiers. The results revealed that these nanoemulsions exhibited optimal behavior when 4% T80, 1% SPI, 2% TS, and 4% SL were used, respectively. Subsequently, we evaluated the stability of the different nanoemulsions. The TS nanoemulsion was found to exhibit the best centrifugal stability and oxidative stability. Additionally, the TS nanoemulsion could maintain good stability after 15 days of storage at 4 °C and 25 °C because of its powerful electrostatic repulsion between droplets. Under different environmental conditions, the TS nanoemulsion also maintained better dispersion and higher OEO encapsulation efficiency than the other nanoemulsions, although it was affected by salt treatment to a certain extent. In summary, TS has a good application prospect as a natural emulsifier for encapsulating bioactive components in the food industry.

## Figures and Tables

**Table 1:** Details about composition of nanoemulsion samples.

Nanoemulsion Samples	Nanoemulsions without OEO			OEO-Nanoemulsions			
	Emulsifier Concentration (%, *w*/*v*)	Aqueous Phase (%, *v*/*v*)	MCT-Oil (%, *v*/*v*)	Emulsifier Concentration (%, *w*/*v*)	Aqueous Phase (%, *v*/*v*)	MCT-Oil (%, *v*/*v*)	OEO (%, *v*/*v*)
T80	0.5–8	95	5	4	95	2.5	2.5
SPI		95	5	1	95	2.5	2.5
TS		95	5	2	95	2.5	2.5
SL		95	5				

**Table 2:** Droplet size, PDI, and zeta potential of the nanoemulsions stabilized with different emulsifier types and concentrations.

Emulsifier Type	Emulsifier Concentration (%, *w*/*v*)	Droplet Size (nm)	PDI	Zeta Potential (mV)
T80	0.5	239.1 ± 4.15 ^a^	0.18 ± 0.02 ^a^	−15.00 ± 0.36 ^a^
	1	205.5 ± 1.59 ^b^	0.18 ± 0.01 ^a^	−16.83 ± 1.63 ^b^
	2	189.1 ± 3.47 ^c^	0.19 ± 0.01 ^a^	−18.87 ± 1.00 ^c^
	4	181.9 ± 1.19 ^d^	0.20 ± 0.01 ^a^	−17.37 ± 0.49 ^b^
	8	180.1 ± 3.44 ^d^	0.20 ± 0.01 ^a^	−18.10 ± 0.10 ^c^
SPI	0.5	324.2 ± 8.58 ^c^	0.21 ± 0.01 ^a^	−34.60 ± 0.36 ^c^
	1	271.5 ± 3.84 ^d^	0.21 ± 0.01 ^a^	−33.40 ± 0.15 ^c^
	2	308.2 ± 8.55 ^cd^	0.17 ± 0.02 ^a^	−30.10 ± 0.66 ^b^
	4	352.8 ± 3.49 ^b^	0.16 ± 0.02 ^a^	−28.67 ± 0.29 ^b^
	8	371.7 ± 8.53 ^a^	0.20 ± 0.01 ^a^	−26.27 ± 0.59 ^a^
TS	0.5	227.5 ± 4.69 ^a^	0.17 ± 0.01 ^a^	−40.23 ± 0.42 ^a^
	1	213.9 ± 4.77 ^b^	0.16 ± 0.01 ^a^	−40.97 ± 3.07 ^a^
	2	206.1 ± 0.92 ^bc^	0.19 ± 0.01 ^a^	−42.57 ± 1.30 ^a^
	4	199.3 ± 2.10 ^c^	0.19 ± 0.01 ^a^	−44.50 ± 0.44 ^a^
	8	197.2 ± 5.16 ^c^	0.19 ± 0.01 ^a^	−43.20 ± 0.52 ^a^
SL	0.5	301.8 ± 7.06 ^a^	0.20 ± 0.01 ^a^	−25.37 ± 0.06 ^a^
	1	266.2 ± 2.52 ^b^	0.17 ± 0.02 ^a^	−26.50 ± 0.26 ^a^
	2	226.6 ± 2.45 ^c^	0.16 ± 0.01 ^a^	−25.27 ± 0.21 ^a^
	4	184.9 ± 1.33 ^d^	0.18 ± 0.01 ^a^	−28.70 ± 0.17 ^b^
	8	178.5 ± 2.62 ^d^	0.21 ± 0.01 ^a^	−29.67 ± 0.08 ^b^

Values expressed as mean± standard deviation. ^a–d^ mean the significant differences (*p* < 0.05) between samples with the same emulsifier type of different concentration.

## Data Availability

Data is contained within the article.
